# Anthropometric Foot Variations in Children: A Cross‐Sectional Study Supporting Sex‐Based Last Design

**DOI:** 10.1002/jfa2.70069

**Published:** 2025-07-31

**Authors:** Manuel Pereira Domínguez, Sara García Blanco, Concepción Paralera‐Morales, Aranza Requena Martínez, María P. Quintana‐Montesdeoca, Aníbal Báez‐Suárez

**Affiliations:** ^1^ Clínica Global Sevilla Spain; ^2^ Mathematics Department University Pablo de Olavide Sevilla Sevilla Spain; ^3^ University Miguel Hernández Elche Spain; ^4^ Mathematics Department University of Las Palmas de Gran Canaria Las Palmas Spain; ^5^ Department of Medical and Surgical Sciences University of Las Palmas de Gran Canaria Las Palmas Spain

**Keywords:** anthropometry, child development, foot, footwear, sex characteristics, three‐dimensional imaging

## Abstract

**Introduction:**

Proper shoe fit is essential for healthy foot development in children. Despite growing interest in the ergonomics of pediatric footwear, scientific evidence on sex‐based foot morphology remains limited. Current unisex shoe lasts may not adequately accommodate anatomical differences between boys and girls, particularly from mid‐childhood onward.

**Methods:**

A cross‐sectional, observational study was conducted involving 1214 school‐aged children (680 boys and 534 girls) between the ages of 1 and 16. Three‐dimensional (3D) foot scans were used to obtain detailed anthropometric data, including toe length, ball area, ball perimeter, and metatarsal dimensions. Sex differences were assessed using independent‐samples t‐tests, and statistical significance was set at *p* < 0.05.

**Results:**

Significant sex‐based differences were found across several key foot dimensions. Boys exhibited larger ball area (mean right foot: 1021.35 vs. 865.85 mm^2^ in girls, *p* < 0.001), greater ball perimeter (207.54 vs. 192.39 mm, *p* < 0.001), and wider and higher metatarsal regions. These differences persisted even when toe length was held constant, suggesting divergent growth patterns in foot morphology. The findings indicate that a single unisex last may not be appropriate for children, especially from shoe size 32 onwards.

**Conclusions:**

This study provides robust anthropometric evidence supporting the need for sex‐specific shoe last design in pediatric populations. Incorporating such data into footwear manufacturing could enhance ergonomic fit, improve comfort, and promote healthy motor development in children. Further longitudinal and multicenter research is needed to establish global standards for pediatric footwear.

**Trial Registration:**

ClinicalTrials.gov identifier: NCT05386992

## 1 Introduction

Humans use footwear to protect their feet from adverse weather conditions and uneven terrain [1]. In the era of science and technology, footwear has evolved into more than just an essential article of clothing; it has become a tool for fostering optimal child development, enhancing occupational and athletic performance, and preventing and treating various pathologies [1, 2].

During childhood, inappropriate footwear hinders proper foot development and optimal mobility, negatively impacting gait acquisition and motor pattern evolution, which may lead to deformities and muscular dysfunctions [2, 3]. Therefore, the influence of footwear on walking and running should be carefully considered by manufacturers to prevent adverse effects associated with its use [3]. The anthropometric characteristics of children's feet differ considerably from those of adult feet; indeed, the structure of the foot is not fully consolidated until the ages of 18–19 years [2]. Thus, it is essential for footwear manufacturers to understand these characteristics to achieve an adequate design [4].

The morphological and functional development of children’s feet is particularly vulnerable to poorly fitting footwear [2, 3]. To design footwear that offers a healthy fit for a wide variety of children, it is crucial to collect accurate data and measurements of children's feet using a large and representative sample [4]. To date, studies collecting such data in our country are scarce [2].

Current scientific evidence underscores the importance of considering the unique morphology of children’s feet, as well as the high functional demands placed on footwear during childhood. There is a notable lack of studies that thoroughly analyze the shape of children’s feet. Previous research has shown that footwear manufacturers often fail to account for the necessary three‐dimensional anthropometric characteristics in their current designs to ensure a healthy fit [5].

To design ergonomic and healthy footwear, it is necessary to consider the biomechanical requirements of children’s feet and conduct an in‐depth analysis of their morphological characteristics and precise anthropometric measurements [5, 6]. Healthy footwear allows for proper foot development and prevents the occurrence of injuries and deformities [6]. Producing healthy footwear requires the creation of lasts based on scientific evidence and rigorously collected data regarding the biomechanics of children’s feet [6]. Given that the currently available data originate from outdated studies with small sample sizes, there is a pressing need to update these variables and transfer the findings to the production sector.

Therefore, the objective of the present research is to conduct an anthropometric and biomechanical analysis of children’s feet to utilize this data in the creation of a standard last that respects their morphology. To this end, we aim to demonstrate that, relying on the ‘know‐how’ of specialized professionals in the field and through a pediatric anthropometric and biomechanical study, it is feasible to design and manufacture lasts that, in turn, enable the creation of healthy children's footwear, thereby improving current models.

## 2 Methods

### 2.1 Study Design

An observational, cross‐sectional, and descriptive study was conducted with the aim of analyzing the anthropometric measurements of the foot in a representative sample of school‐aged boys and girls. The research was carried out following the guidelines of the Declaration of Helsinki and was approved by the Ethics Committee of the Virgen Macarena ‐ Virgen del Rocío University Hospitals of Seville (Spain) under reference code 0542‐N‐22. Additionally, the study was registered on ClinicalTrials.gov (NCT05386992). All legal representatives of the participants signed the informed consent beforehand.

### 2.2 Participants

A total of 1214 boys and girls (2428 registered samples) enrolled in various educational institutions in the Seville region (Spain) were randomly selected. The selection of schools and participants within each institution was performed through simple random sampling, ensuring the representativeness of the pediatric population. The inclusion criteria were

- School‐age boys and girls with foot sizes ranging from 20 to 41.
- Enrolled in public, private, or charter schools to ensure sample variability regarding the type of educational institution.

The exclusion criteria were

- The presence of a severe structural or functional foot alteration that prevents proper digitization of the foot and ankle using the hardware and software employed for data collection.
- Lack of signed informed consent and explicit authorization from parents or legal guardians for measurement and subsequent data processing.
- Inability to stand or walk independently.
- Displaying crying, fear, noncooperative behavior, or engaging in inappropriate conduct.

### 2.3 Sample Size

The sample size calculation was conducted considering that the main hypothesis of our study is to estimate the population mean through hypothesis testing. For this estimation, we focused on the length of the foot (Fl), which is the most important variable in the study.

The calculation will be performed using the following equation [7]:

$$n=\frac{{\left(Z_{1-\frac{\alpha }{2}}+Z_{1-\beta }\right)}^{2}}{\frac{d^{2}}{\sigma_{0}^{2}}}$$

where *α* represents the Type I error, *β* the Type II error, 1‐*β* the power of the test, *d* the minimum detectable difference, and σ02\sigma_0^2 the estimated population variance.

A 95% confidence level and an 80% power were used. The population variance estimation was obtained from a previously conducted pilot study. The minimum detectable difference *d* considered is 0.5 mm. This estimation was carried out by segmenting according to foot size (half sizes).

Losses/dropouts/withdrawals from the sample: Based on the initial pilot study and our clinical experience with this type of patients, we estimated a data loss of less than 15%, leading to a required sample size of 1200.

### 2.4 Procedure

An anthropometric study of the feet of the subjects included in the sample was conducted. The variables of interest used in this study were collected through the digitization of each participant's foot and ankle, as well as through an anonymous questionnaire completed and submitted by the study volunteers and their parents or legal guardians.

**FIGURE 1:**
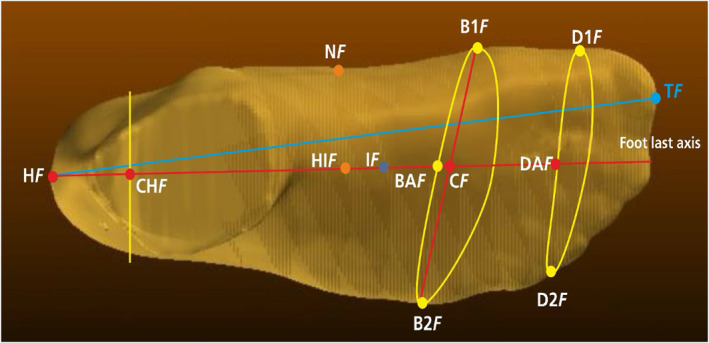
Point cloud representation and mesh generation from a 3D foot scan. Foot reference points. B1F, medial metatarsal point; B2F, lateral metatarsal point; BAF, underside ball midpoint; CF, mid metatarsal point; CHF, heel seat center; DAF, underside toes midpoint; HF, back point; HF‐TF, foot aligned length; HIF, high instep point; IF, instep point; NF, navicular point.

**FIGURE 2:**
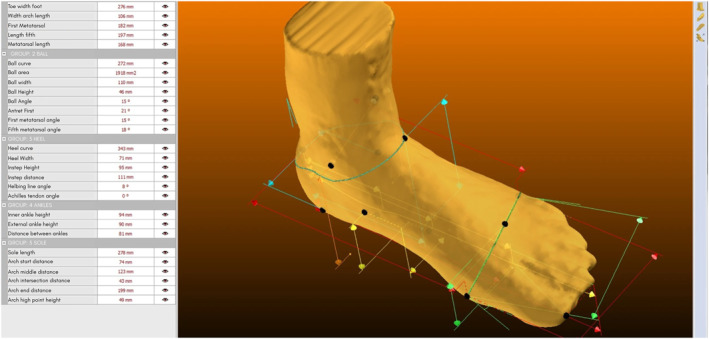
Example of a 3D foot scan mesh with extracted anthropometric variables.

A three‐dimensional (3D) foot scanner (Icad PIE, INESCOP, Spain) was utilized to obtain detailed morphological data of the foot. The device operates by projecting a sequence of infrared laser dot lines, which are subsequently detected by dedicated sensors. The system determines the time delay between the emission and reception of the laser beams, allowing for precise measurements along the foot's surface. The system captures a full 3D image in approximately 18 s with a spatial resolution of 1 mm. Upon completing the full scan, the collected data are represented as a point cloud, from which a mesh can be generated (Figure 1). The mesh serves as a reference for extracting specific anthropometric measurements using the scanner’s accompanying software (Figure 2). Additionally, to enhance the accuracy of the obtained images, a filtering process is applied to eliminate potential noise caused by ambient light.

**FIGURE 3:**
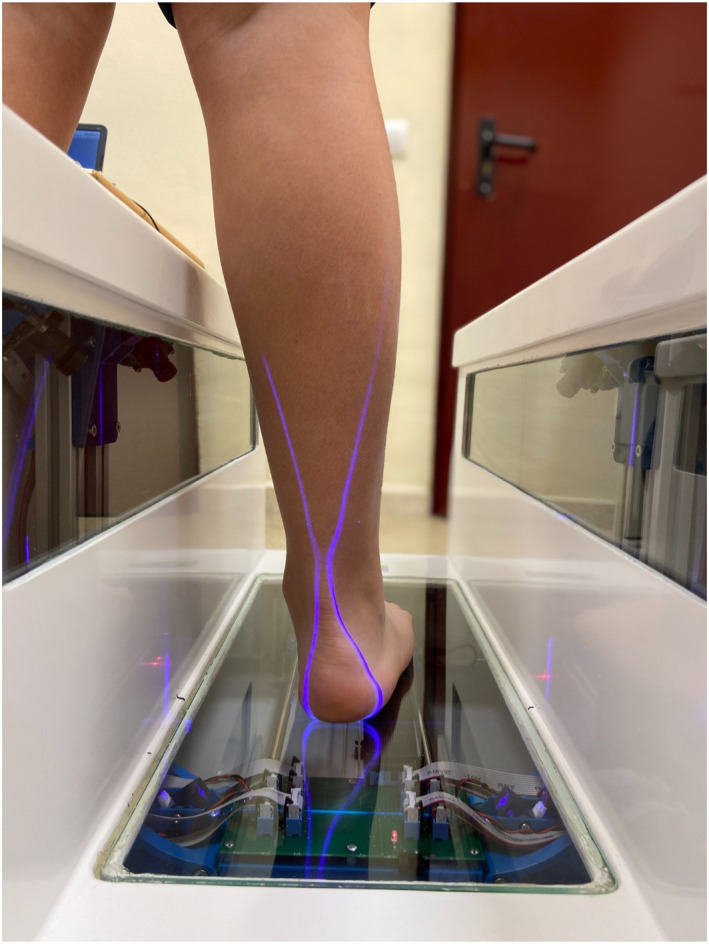
Foot evaluation procedure.

For accurate measurement and to ensure the replication of the procedure, the protocol to be followed consisted of the following steps: The participant stands in an orthostatic position on the 3D scanner, with hips, knees, and ankles in a neutral position (Figure 3). It is crucial that the ankle remains at 90° and that the participant remains still during the test to prevent errors in data collection. One foot is placed in the designated scanning area, whereas the other is positioned on an adjacent external surface, acting as a step. It is indifferent whether the left or right foot is scanned first, as this does not affect the final test result.

The device was calibrated monthly, following the manufacturer's protocol. Although no new reliability analysis was conducted in this study, the Icad PIE system has demonstrated high consistency in previous validations.

This methodological approach complies with the CRITIC reporting guidelines for studies using 3D foot scanning [8]. Details regarding the extracted anthropometric variables are presented in the Data Collection section. All updates made in response to reviewer comments have been highlighted in yellow in the revised manuscript.

### 2.5 Data Collection

In addition to baseline data related to age, sex, height, weight, and BMI, the anthropometric variables primarily focused on measuring the toe length and ball area as key data points. A comprehensive analysis was then conducted, comparing the right and left foot, as well as potential differences between sexes.

Furthermore, the following foot dimensions were recorded: foot width, arch length, first metatarsal length, fifth metatarsal length, anatomical ball perimeter, ball width, ball height, ball angle, first metatarsal angle, fifth metatarsal angle, heel perimeter‐instep height, heel width, instep height, Helbing's line angle, plantar length, navicular height, and standard ball perimeter.

### 2.6 Data Analysis

The data analysis was performed using the statistical software IBM SPSS 27 (IBM Corp. Released 2020. IBM SPSS Statistics for Windows, Version 27.0. Armonk, NY: IBM Corp). Absolute frequencies and relative percentages were used to summarize categorical variables. The equality of proportions for dichotomous categories was tested using the nonparametric binomial test. To assess the potential association between two categorical variables, Fisher's exact test was employed. Numerical variables were summarized using the mean and standard deviation (SD), as well as the minimum and maximum values for each. To analyze the potential association between two numerical variables, Pearson's linear correlation coefficient test was used. To assess differences between sexes and between foot sides (right/left) for continuous anatomical variables, we applied generalized estimating equation (GEE) to correct for the lack of independence between paired foot measurements. GEE models used an exchangeable correlation structure and clustered observations by participant ID [9]. Sex and foot side were included as fixed effects in each model. In addition, effect sizes (*Cohen's d*) were calculated to quantify the magnitude of sex differences, following the cutoffs proposed by Sawilowsky [10]. Results were considered statistically significant if *p* < 0.05.

## 3 Results

In this cross‐sectional study, a total of 1214 schoolchildren (680 boys and 534 girls) aged between 1 and 16 years participated. Both feet were measured for each child, resulting in a total of 2428 samples. The mean age of the sample was 7.53 years (SD = 3.58), and the mean BMI was 19.22 (SD = 3.65 kg/m^2^) in boys and 17.48 (SD = 2.68 kg/m^2^) in girls. The difference between sexes was not statistically significant (*p* = 0.735) (Table 1).

**TABLE 1:** Descriptive for age, weight, height, and BMI of the entire sample by gender.

		Total				Male				Female		
	Mean	95% CI		SD	Mean	95% CI		SD	Mean	95% CI		SD
BMI (kg/m2)	19.22	17.33	17.63	2.68	17.59	17.38	17.79	2.69	17.35	17.12	17.57	2.66
Age (year)	7.53	7.33	7.73	3.58	7.75	7.46	8.03	3.75	7.25	6.96	7.53	3.32
Weight (kg)	28.56	27.86	29.26	8.97	29.19	28.23	30.16	12.86	27.75	26.75	28.75	11.73
Height(cm)	124.69	123.42	125.96	22.58	125.52	123.77	127.28	23.30	123.64	121.80	125.48	21.61

The results show quantifiable differences in foot measurements between boys and girls (Table 2). In terms of toe length (FL), boys present higher values with a mean of 213.57 ± 35.04 mm in the right foot and 213.73 ± 35.42 mm in the left foot, compared to girls, who show means of 198.49 ± 34.18 mm and 198.82 ± 33.88 mm, respectively (*p* < 0.001). The anatomical perimeter of the metatarsal (Bc) is also greater in boys, with values of 208.07 ± 31.28 mm in the right foot and 207.42 ± 31.16 mm in the left foot, whereas girls recorded means of 193.76 ± 40.69 mm and 194.34 ± 30.17 mm, respectively (*p* < 0.001).

**TABLE 2:** General foot measurements by gender.

Measurement (unit)	Female (mean ± SD)	Male (mean ± SD)	*p*‐value	Cohen's d
Toe length (mm)	198.65 ± 34.03	213.65 ± 35.23	< 0.001	0.43
Anatomical perimeter (mm)	194.05 ± 35.43	207.74 ± 31.22	< 0.001	0.41
Metatarsal area (mm^2^)	863.15 ± 285.56	1017.60 ± 339.78	< 0.001	0.49
Metatarsal width (mm)	77.72 ± 11.41	84.11 ± 12.19	< 0.001	0.54
Metatarsal height (mm)	28.76 ± 3.34	30.20 ± 3.49	< 0.001	0.42
Metatarsal angle (°)	12.55 ± 4.00	12.54 ± 3.54	0.919	−0.003
Standard perimeter (mm)	191.95 ± 26.06	207.00 ± 28.27	< 0.001	0.55

Regarding the metatarsal area (Ba), boys exhibit means of 1021.35 ± 343.60 mm^2^ in the right foot and 1013.84 ± 335.97 mm^2^ in the left foot (Table 3), values significantly higher than those of girls (865.85 ± 279.02 mm^2^ and 860.45 ± 292.10 mm^2^, respectively, *p* < 0.001). Similarly, the metatarsal width (Bw) is greater in boys (83.93 ± 12.18 mm in the right foot and 84.30 ± 12.19 mm in the left foot) compared to girls (77.55 ± 11.42 mm and 77.90 ± 11.39 mm, respectively, *p* < 0.001).

**TABLE 3:** Comparison of foot measurements by gender and laterality.

Measurement (Unit)	Foot	Female (mean ± SD)	Male (mean ± SD)	*p*‐value
Toe length (mm)	Right	198.49 ± 34.18	213.57 ± 35.04	< 0.001
Toe length (mm)	Left	198.82 ± 33.88	213.73 ± 35.42	< 0.001
Anatomical perimeter (mm)	Right	193.76 ± 40.69	208.07 ± 31.28	< 0.001
Anatomical perimeter (mm)	Left	194.34 ± 30.17	207.42 ± 31.16	< 0.001
Metatarsal area (mm^2^)	Right	865.85 ± 279.02	1021.35 ± 343.60	< 0.001
Metatarsal area (mm^2^)	Left	860.45 ± 292.10	1013.84 ± 335.97	< 0.001
Metatarsal width (mm)	Right	77.55 ± 11.42	83.93 ± 12.18	< 0.001
Metatarsal width (mm)	Left	77.90 ± 11.39	84.30 ± 12.19	< 0.001
Metatarsal height (mm)	Right	29.04 ± 3.29	30.49 ± 3.42	< 0.001
Metatarsal height (mm)	Left	28.48 ± 3.38	29.92 ± 3.56	< 0.001
Metatarsal angle (°)	Right	11.33 ± 3.81	11.34 ± 3.61	0.938
Metatarsal angle (°)	Left	13.77 ± 4.12	13.73 ± 3.47	0.899
Standard perimeter (mm)	Right	192.39 ± 25.94	207.54 ± 28.41	< 0.001
Standard perimeter (mm)	Left	191.52 ± 26.18	206.46 ± 28.14	< 0.001

In terms of metatarsal height (Bh), significant differences were observed, with boys showing higher values in both feet (30.49 ± 3.42 mm in the right and 29.92 ± 3.56 mm in the left), whereas girls had means of 29.04 ± 3.29 mm and 28.48 ± 3.38 mm, respectively (*p* < 0.001). However, the metatarsal angle (Ban) does not present significant differences between sexes (*p* > 0.05), with mean values of 11.33 ± 3.81° in girls and 11.34 ± 3.61° in boys for the right foot and 13.77 ± 4.12° and 13.73 ± 3.47° for the left.

The standard perimeter of the metatarsal (Bs) was consistently higher in boys, with values of 207.54 ± 28.41 mm in the right foot and 206.46 ± 28.14 mm in the left, whereas girls recorded values of 192.39 ± 25.94 mm and 191.52 ± 26.18 mm, respectively (*p* < 0.001).

Statistically significant sex differences were found in all anatomical measurements except for the metatarsal angle. Cohen's *d* values ranged from 0.41 to 0.55 for these differences, indicating moderate effect sizes in toe length, perimeter, area, width, and height of the foot. The metatarsal angle did not differ between sexes (*p* = 0.919, *d* = −0.003), suggesting that the angular alignment is similar in boys and girls.

The variable was dichotomized based on foot size, with measurements classified as ≤ 32 or > 32. The overall mean foot size in the sample was 26.47 (SD = 3.84) for sizes ≤ 32, and 36.75 (SD = 2.89) for participants with foot sizes > 32.

**FIGURE 4:**
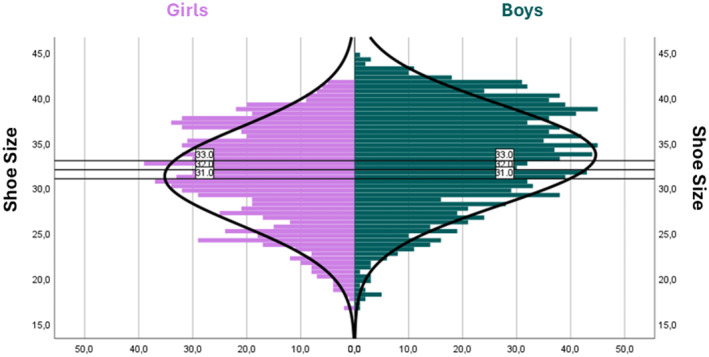
Histogram representation shows the sample as a function of foot length and distributed by gender.

Regarding sex distribution, the mean foot size was 33.65 (SD = 5.54) in boys and 31.28 (SD = 5.36) in girls. A histogram was created to represent the entire sample based on foot length. Three reference lines were included for sizes 31, 32 (206.66 mm), and 33, which served as the center of gravity for illustrating the Gaussian bell curve (Figure 4).

Additionally, a percentile analysis was performed using size 31 (Table 4) and size 32 (Table 5) as references. Statistically significant differences were observed (*p* = 0.004) when using a foot size of 32 as the cutoff point, with median values recorded at 28.400 (boys) and 27.800 (girls). In this way, discrepancies in foot size between boys and girls were evident in sizes > 32.

**TABLE 4:** Percentile table oriented by shoe size 31 as a reference.

	Percentile						
Gender	**5**	**10**	**25**	**50**	**75**	**90**	**95**
Male	32,600	33,200	34,600	37,100	39,700	41,400	42,135
Female	32,500	32,800	33,900	35,800	37,700	39,100	40,200

*Note:* Values in bold indicate foot length (cm) at each percentile of the studied children’s distribution (e.g., the 50th percentile represents the median, where 50% of children have smaller foot length and 50% larger).

**TABLE 5:** Percentile table oriented by shoe size 32 as a reference.

	Percentile						
Gender	**5**	**10**	**25**	**50**	**75**	**90**	**95**
Male	21,665	23,300	25,700	28,400	30,500	31,490	31,745
Female	20,505	21,910	24,325	27,800	30,200	31,200	31,700

*Note:* Values in bold indicate foot length (cm) at each percentile of the studied children’s distribution (e.g., the 50th percentile represents the median, where 50% of children have smaller foot length and 50% larger).

Generalized estimating equation (GEE) models were used to assess differences in foot morphology by sex and laterality while adjusting for within‐subject correlations. Results indicated that all measured parameters were significantly different between boys and girls (*p* < 0.001 in all models), with boys showing consistently larger values in foot length, anatomical perimeter, metatarsal area, width, and height (Table 6). No significant differences were observed between left and right feet in most variables, except for metatarsal height (*p* = 0.0011) and metatarsal angle (*p* < 0.001), where the left foot showed slightly lower values.

**TABLE 6:** GEE model results for foot morphology variables.

Morphological variable	Coefficient (sex: Girl vs. boy)	*p*‐value (sex)	Coefficient (side: Left vs. right)	*p*‐value (side)
Foot length (FL)	−14.997	*p* < 0.001	0.234	*p* = 0.8750
Anatomical perimeter (Bc)	−13.696	*p* < 0.001	−0.117	*p* = 0.9340
Metatarsal area (Ba)	−154.438	*p* < 0.001	−6.595	*p* = 0.6270
Metatarsal width (Bw)	−6.390	*p* < 0.001	0.363	*p* = 0.4730
Metatarsal height (Bh)	−1.416	*p* < 0.001	−0.516	*p* = 0.0011
Metatarsal angle (Ban)	−1.998	*p* < 0.001	−1.307	*p* < 0.001
Standard perimeter (Bs)	−13.592	*p* < 0.001	−0.402	*p* = 0.7210

## 4 Discussion

The results of this cross‐sectional study clearly confirm significant anthropometric differences in foot morphology between boys and girls, particularly evident from shoe size 32 onwards. The findings indicate that boys’ feet are wider in the midfoot regions, consequently resulting in greater overall length and size compared to girls. This supports the conclusion that scaling a unisex last for these sizes is not appropriate.

Furthermore, it was observed that, under equal conditions of effective first toe length, higher percentiles in boys are associated with notable increases in anthropometric parameters such as ball area and ball perimeter. These findings underscore that foot growth is neither proportional nor symmetrical across all regions, which suggests that the scaling of children's footwear lasts should explicitly account for these specific morphological traits [11].

Importantly, the statistical analysis accounted for the correlation between left and right feet within subjects using a generalized estimating equation (GEE) model. This approach confirmed significant sex‐related differences in all morphological variables while showing no relevant asymmetry between the two feet, except in the metatarsal height and angle. These findings support the robustness of our results and their relevance for sex‐specific and morphology‐adapted footwear design.

These findings are consistent with previous research emphasizing the importance of adapting children’s footwear to their specific anatomical characteristics to prevent potential biomechanical and pathological alterations associated with inadequate last design, such as pes planovalgus or cavus feet [4]. These conditions can significantly alter anthropometric measurements [1, 12]. Therefore, it is essential that footwear manufacturers rely on such anthropometric data to optimize their designs and provide ergonomic products that support healthy foot development and prevent musculoskeletal complications [4].

This study identified a significant increase in the ball area in boys compared to girls, even after controlling for toe length. Similarly, the ball perimeter showed substantial sex‐related differences, being consistently greater in boys across all measurements. These findings align with existing literature that highlights the importance of incorporating detailed anthropometric data in the production of children's footwear, since failure to do so may adversely affect both foot development and gait biomechanics [2, 3].

In addition, differences identified in other anthropometric variables, such as metatarsal width and height, further support the study’s initial hypothesis, reinforcing the need for sex‐specific adaptations in footwear design. Previous studies have indicated that variability in these parameters is critical to ensuring proper load distribution and pressure patterns during walking and running, which are essential for healthy motor development [5, 11].

Nevertheless, this study faces several important limitations. Chief among them is the scarcity of robust scientific literature specifically addressing sex‐differentiated anthropometric foot measurements in children, as well as a lack of objective criteria for defining the essential features of healthy children's footwear. Much of the currently available information derives from nonscientific sources, contributing to significant variability and difficulty in standardizing manufacturing protocols.

Another relevant limitation is the study’s cross‐sectional design and its geographically localized setting in Seville, Spain, which restricts the generalizability of the findings to other populations with differing racial, cultural, or socioeconomic characteristics [6]. Although the sample size exceeds 1000 participants, increasing it could further enhance the representativeness and global applicability of the results. Additionally, technical limitations were observed in the current 3D scanning systems, which present errors in data acquisition and interpretation, particularly for smaller shoe sizes. This highlights the need for further development and refinement of scanning software to improve measurement precision in future studies.

The clinical and technological translation of these findings is of considerable importance, as they offer a solid scientific foundation for improving the design of children’s footwear. This may facilitate the unification of criteria within the traditional artisanal footwear sector. Incorporating these findings into industrial processes could reduce the incidence of pediatric foot pathologies, improve comfort, and optimize motor development [2, 5].

For future research, we recommend the implementation of longitudinal and multicenter studies to assess the long‐term impact of adapted footwear on pediatric foot development. Furthermore, efforts should be made to promote the international standardization of specific anthropometric measurements and ergonomic footwear design criteria, considering additional variables such as habitual physical activity, commonly worn footwear types, and lifestyle characteristics across diverse populations.

## 5 Conclusions

In conclusion, this study provides strong evidence supporting the need to differentiate children’s footwear design by sex from shoe size 32 onwards, based on significant anthropometric differences—particularly in the ball area and perimeter. These results have direct implications for the footwear industry, underscoring the necessity of sex‐specific lasts adapted to the differential growth patterns of boys and girls. Future research should focus on longitudinal and multicenter studies to further evaluate the long‐term effects of tailored footwear on children's physical development.

## Author Contributions

**Manuel Pereira Domínguez:** conceptualization (lead), writing – original draft (supporting), writing – review and editing (equal), validation (lead). **Sara García Blanco:** methodology (lead), writing – review and editing (equal). **Concepción Paralera‐Morales:** data curation (lead), review and editing (equal). **Aranza Requena Martínez:** resources (lead), writing – review and editing (equal). **María P. Quintana‐Montesdeoca:** formal analysis (lead), validation (supporting). **Aníbal Báez‐Suárez:** conceptualization (supporting), writing – original draft (lead), writing – review and editing (lead).

## Ethics Statement

This study was conducted in accordance with the Declaration of Helsinki and was approved by the Ethics Committees of the Research Ethics Committee of the Virgen Macarena ‐ Virgen del Rocío University Hospitals of Seville. (Spain) (Code 0542‐N‐22).

## Consent

The parents were previously informed about the characteristics of the study. They were all asked to complete a questionnaire and to provide signed consent to confirm the participation of their children in the study.

## Conflicts of Interest

The authors declare no conflicts of interest.

## Data Availability

The data that support the findings of this study are available from the corresponding author upon reasonable request.
